# Predictive Factors for Drain Placement After Laparoscopic Cholecystectomy

**DOI:** 10.3389/fsurg.2021.786158

**Published:** 2022-02-02

**Authors:** Giacomo Calini, Pier Paolo Brollo, Rosanna Quattrin, Vittorio Bresadola

**Affiliations:** ^1^Department of Medicine, General Surgery Department and Simulation Center, Academic Hospital of Udine, University of Udine, Udine, Italy; ^2^Department of Organization of Hospital Services, Academic Hospital of Udine, Udine, Italy

**Keywords:** preliminary study, predictive factor, laparoscopic cholecystectomy, drain placement, retrospective study

## Abstract

**Purpose:**

Currently, surgical drainage during a laparoscopic cholecystectomy (LC) is still placed in selected patients. Evidence of the non-beneficial effect of the surgical drain comes from studies with a heterogeneous population. This preliminary study aims to identify any clinical, demographic, or intraoperative predictive factors for a surgical drain placement during LC as the first step to identify population for a prospective randomized study.

**Method:**

The study was conducted in a single referral center and academic hospital between 2014 and 2018. Patients who underwent unconverted LC were divided into two groups: Group A (drain) and Group B (no drain). We explored baseline, preoperative, intraoperative characteristics, and postoperative outcomes.

**Results:**

Between 409 patients who underwent LC: 90 (22%) patients were in Group A (drain). Age >64 years, male sex, cholecystitis, Charlson comorbidity index (CCI) ≥ 1, experienced surgeon, intraoperative technical difficulties, need for an additional trocar, operative time >60 min, and estimated blood loss >10 ml were predictive factors at univariate analysis. While at multivariate analysis, cholecystitis (odds ratio [OR]: 2.8, 95% *CI*:1.5–5.1; *p* < 0.001), CCI ≥ 1 (*OR*:1.9, 95% *CI*:1.0–3.5; *p* = 0.05), intraoperative technical difficulties (*OR*: 3.6, 95% *CI*:1.8–6.2; *p* < 0.001), need of an additional trocar (*OR*: 2.5, 95% *CI*: 1.4–4.4; *p* < 0.005), and estimated blood loss >10 ml (*OR*: 3.0, 95% *CI*:1.7–5.3; *p* < 0.0001) were predictive factors for a surgical drain placement during LC.

**Conclusions:**

This study identified predictive factors that currently drive the surgeons to a surgical drain placement after LC. Randomized prospective studies are needed to define the use of drain placement in these selected patients.

## Introduction

In the past decades, cholecystectomy has become one of the most frequently performed surgical procedures, both in the elective and in the urgency/emergency setting (1). The laparoscopic approach is now the first choice and in most cases, it allows to complete the surgical procedure with excellent patient outcomes, adding the laparoscopic advantages in terms of postoperative management (2–4). The usefulness of surgical drain placement after a cholecystectomy has long been debated. Although numerous randomized clinical trials and meta-analyses have already clearly shown that the use of surgical drain does not improve the postoperative outcomes of patients, surgical drains are still used in selected populations (5–7). Over the years, the evolution of surgical technique and the increasing life expectancy of the general population has led to an expansion of surgical indications to a wider and heterogeneous pool of patients (8–12). The progress of surgical technique, the increasing experience of operators, and the accumulation of scientific evidence greatly influenced a more restricted use of surgical drain at the end of a cholecystectomy (13). However, it still remains a device used in conditions that often raise serious concerns about its usefulness or even raise doubts about its potentially harmful effects. These considerations are particularly relevant for patients who are more fragile for clinical and age-related reasons, such as the elderly (4, 14). To date, the evidence of the non-beneficial effect of the surgical drain comes from studies with a heterogeneous population (5, 6, 15, 16). We hypothesize that the intraoperative drain placement might still have a role in selected populations. Hence, defining the predictive factors for surgical drain placement in the current practice is the first step to identify the population for a prospective randomized study. In light of the above, the aim of this preliminary study was to identify any predictive factors for the intraoperative drain placement in patients undergoing laparoscopic cholecystectomy (LC).

## Methods

This study was conducted in an Italian Academic Hospital (AH) from March 2020 to September 2020. It was consisted of a retrospective analysis of hospital discharge data regarding all patients who underwent LC, identified by the International Classification of Diseases, Ninth Revision, Clinical Modification (ICD-9-CM) code 51.23, as the primary procedure not associated with any other significant surgery during the 2014–2018 period. Data were collected, anonymously and in aggregate form, from the Regional Socio-Health Information System (SISSR) and analyzed by extracting information about the demographic characteristics of patients, urgency and diagnosis upon admission to the hospital, surgery procedure, perioperative course, comorbidities, and complications using ICD-9-CM codes. In the second step, two experienced surgeons reviewed all cases to exclude those with malignant biliary disease and with cholelithiasis complicated by acute pancreatitis or obstruction of the common bile duct. After local IRB approval of the study, hospital clinical computerized records were consulted to implement information not available in the regional database.

Patients were allocated into two groups: Group A (patients with surgical drain placement during LC) and group B (patients without drain placement). Both groups of patients were investigated with respect to the following variables: (1) general characteristics: age, sex, body mass index (BMI) (underweight: <18.5; normal weight 18.5–24.9; overweight 25.0–29.9; and obese ≥30.0); (2) preoperative clinical conditions: severity of cholelithiasis, comorbidity (by using ICD-9-CM codes) and Charlson comorbidity index (CCI) with no age-adjustment (17); (3) surgical procedure: admission urgency (elective and non-elective), first surgeon (experienced surgeon with more than 10-year laparoscopic experience and resident), intraoperative technical difficulties described in the surgical report (defined as fibrotic adherence, intentional puncture to decompress the gallbladder, gallbladder rupture with bile contamination of the operating field, bleeding from the cystic artery, difficult individuation of the anatomical structures, liver steatosis/cirrhosis, and subtotal cholecystectomy), need for an additional trocar, operating time, and estimated blood loss. The ICD-9-CM diagnosis codes used to classify comorbidities related to cholecystectomy are reported in the Supplementary Material. CCI was grouped into 3 classes: 0, 1–2, and ≥3; we used the CCI without age to avoid the collinearity in multivariate analysis. The clinical severity of cholelithiasis was divided into two categories: (1) cholelithiasis only (ICD-9-CM codes: 574.20, 574.50, 574.90, 574.21, and 575.3) and (2) cholecystitis with or without cholelithiasis (ICD-9-CM codes: 574.10, 574.40, 574.70, 575.1, 574.00, 575.0, 575.11, and 575.12). The events considered as predictors of negative clinical outcome occurring in the two groups after LC were: (1) postoperative complications, as described in the postoperative notes, discharge summary, and post-discharge follow-up/imaging (bile leak, intra-abdominal fluid, wound complications, and incisional hernia), and their severity level expressed by the Clavien-Dindo classification (CDC) (18); (2) surgical re-intervention within 30 days; and (3) increase in the postoperative hospital stay.

In our center, LC has been performed according to a standardized technique (19). The patient is positioned in stirrups with the primary operator standing between the legs. A blunt umbilical trocar is positioned with an open technique. After exploration of the peritoneal cavity, other 2–3 trocars are inserted under vision. The dissection of the Calot's triangle is performed to reach the “critical view of safety” with the aim to identify and dissect the cystic duct and cystic artery. Clipping and division of the structures are then carried out. Intraoperative cholangiography and gallbladder needle decompression are not routinely performed. Retrograde dissection of liver bed is completed and the gallbladder removed by using an endobag. An abdominal surgical drain is not routinely placed; when it does, we place an 18 Fr tubular close drain (19).

All analyses were performed using the statistical software SPSS, V.20. The description of the data was done in the form of mean and SD for quantitative data and frequency and proportion for qualitative data. Age was described both as a continuous variable and as a qualitative variable. The population was divided into elderly (≥65 years) and non-elderly (<65 years). The first analysis of the data was done to test statistically significant differences between the two groups of patients, Group A (with surgical drain) and Group B (without drain placement), with regard to preoperative, intra-operative, and postoperative variables. An independent *t*-test for quantitative data and chi-square test for qualitative ones were used. The second endpoint was to explore the predictive factors that supported the decision of surgeon to insert a drain after LC. Univariate and multivariate analyses with odds ratios (ORs) and 95% CIs were used. The choice of potential predictive factors for the multivariate model was based on the results of univariate analysis, with *p* < 0.05 as the criterion for inclusion. The factors that did not maintain an association in the final model were considered not to be associated after correction by the confounding factors. Statistical significance was set at a *p* < 0.05.

## Results

In total, 409 patients underwent LC as the primary procedure during hospitalization in our AH between 2014 and 2018. An intraoperative surgical drain was placed in 90 patients (Group A) and was not placed in 319 patients (Group B). Preoperative and operative data in both groups are shown in Table 1. In regard to the preoperative data, group A was found to be statistically older (44.4 vs. 26.0%, p < 0.001), with a higher incidence of cholecystitis (52.2 vs. 21.3, p < 0.0001) and with a CCI (with no age) ≥1 (35.6 vs. 19.4, p < 0.01). There were statistically significant differences between the two groups in all operative data: group A presented a longer operative time (112.6' + 30.0' vs. 77.8' + 27.4', p < 0.0001), more intraoperative technical difficulties (77.8 vs. 42.3%, p < 0.0001), need of an additional trocar (50.0 vs. 21.9%, p < 0.0001), and estimated blood loss >10 ml in more patients (68.9 vs. 36.4%, p < 0.0001), as compared with group B. Among the postoperative outcomes of interest in Table 2, group A and group B had a statistically significant difference in the occurrence of postoperative surgical complications (18.9 vs. 8.8%, p < 0.01), overall postoperative complications (28.9 vs. 14.7%, p < 0.01) and mean postoperative hospital length of stay (2.3 + 2.1 vs. 1.2 + 0.7 days, p < 0.0001).

**Table 1:** Pre- and intra-operative data for patients undergoing laparoscopic cholecystectomy (LC).

	**Group A (drain) (*n* = 90)**	**Group B (no drain) (*n* = 319)**	***p-*value**
**DEMOGRAPHIC AND BASELINE CHARACTERISTICS**			
**Age**			
0–64 years	50 (55.6)	236 (74.0)	* <0.001*
>64 years	40 (44.4)	83 (26.0)	
Mean (SD)	59.8 (14.8)	52.7 (15.2)	
**Sex**			
Male	44 (48.9)	120 (37.6)	*0.05*
Female	46 (51.1)	199 (62.4)	
**BMI**			
<18.5	–	4 (1.3)	*0.07*
18.5–24.9	13 (14.4)	95 (33.9)	
≥25	68 (75.6)	207 (64.9)	
*Missing*	*9 (10.0)*	*13 (4.1)*	
**Severity of cholelithiasis**			
Cholelithiasis only	43 (47.8)	251 (78.7)	* <0.0001*
With cholecystitis	47 (52.2)	68 (21.3)	
**Comorbidity ICD-9-CM codes** ^**a**^			
Present	12 (13.3)	36 (11.3)	*0.6*
Absent	78 (86.7)	283 (88.7)	
**Charlson comorbidity index (without age)**			
0	58 (64.4)	257 (80.6)	* <0.01*
1–2	27 (30.0)	54 (16.9)	
≥3	5 (5.6)	8 (2.5)	
**Hospital admission**			
Urgent	19 (21.1)	58 (18.2)	*0.5*
Planned	71 (78.9)	261 (81.8)	
**SURGICAL CHARACTERISTICS**			
**First operator**			
resident surgeon	52 (57.8)	237 (74.3)	* <0.005*
experienced surgeon	38 (42.2)	80 (25.1)	
Missing	–	2 (0.6)	
**Intraoperative technical difficulties** ^**b**^			
Present	70 (77.8)	135 (42.3)	* <0.0001*
Absent	20 (22.2)	184 (57.7)	
**Need of an additional trocar**			
Yes	45 (50.0)	70 (21.9)	* <0.0001*
No	45 (50.0)	247 (77.4)	
Missing	–	2 (0.6)	
**Operative time (min)**			
Mean (SD)	112.6 (30.0)	77.8 (27.4)	* <0.0001*
**Estimated blood loss**			
≤10 ml	28 (31.1)	203 (63.6)	* <0.0001*
>10 ml	62 (68.9)	116 (36.4)	

*SD, standard deviation; BMI, body mass index*.

a *Described in Table 1*.

b *Defined as fibrotic adherence, intentional puncture to decompress the gallbladder, gallbladder rupture with bile contamination of the operating field, bleeding from the cystic artery, difficult individuation of the anatomical structures, liver steatosis/cirrhosis, and subtotal cholecystectomy*.

**Table 2:** Postoperative outcomes.

	**Group A (drain) (*n* = 90)**	**Group B (no drain) (*n* = 319)**	***p-*value**
**Overall postoperative complications**	**26 (28.9)**	**47 (14.7)**	*** <0.01***
CDC 1	13 (14.4)	32 (10)	
2	7 (7.8)	9 (2.8)	
3	6 (6.7)	6 (1.9)	
No complications	64 (71.1)	268 (84.3)	
Missing	–	3 (0.9)	
**Surgical postoperative complications**	**17 (18.9)**	**28 (8.8)**	*** <0.05***
Bile leak	1/17 (5.9)	–	
Indra-abdominal fluid collection	4/17 (23.5)	1/28 (3.6)	
Wound complication	10/17 (58.8)	24/28 (85.7)	0.1
Incisional hernia	2/17 (11.8)	3/28 (10.7)	
**Re-operation 30-day**	**3 (3.3)**	**6 (1.8)**	***0.1***
**Postoperative stay (days)** mean (+SD)	**2.3 (+2.1)**	**1.2 (+0.7)**	*** <0.0001***

*CDC, Clavien-Dindo classification; SD, standard deviation.*

Univariate analysis for the predictive factors that drove the decision of surgeon to place the drain after a cholecystectomy is reported in Table 3. Predictive variables were age > 64 years (OR = 2.3; 95% CI: 1.4–3.7; p < 0.0001), male sex (OR = 1.6; 95% CI: 1.0–2.5; p < 0.05), cholecystitis with or without cholelithiasis (OR = 4.0; 95% CI: 2.5–6.6; p < 0.0001), CCI (with no age) value ≥1 (OR = 2.3; 95% CI: 1.4–3.8; p < 0.01), experienced surgeon as first operator (OR = 2.2; 95% CI: 1.3–3.5; p < 0.005), the presence of an intraoperative technical difficulty (OR = 4.8; 95% CI: 2.7–8.2; p < 0.0001), the need of an additional trocar (OR: 3.5, 95% CI: 2.1–5.8; p < 0.0001), operative time >60 min (13.6, 95% CI: 4.2–44.1; p < 0.0001) and estimated blood loss >10 ml (OR: 3.9, 95% CI: 2.3–6.4; p < 0.0001).

**Table 3:** Univariate and multivariate analysis for drain placement after LC.

	**Total LC** **(n = 409)**	**Group A** **(n = 90)**	**Univariate** **OR (95%CI)**	**p-value**	**Multivariate** **OR (95%CI)**	**p-value**
**Age**						
0–64 years	286 (69.9)	50 (17.5)	–			
>64 years	123 (30.1)	40 (32.5)	2.3 (1.4–3.7)	<0.0001		
**Sex**						
Male	164 (40.1)	44 (26.8)	1.6 (1.0–2.5)	0.05		
Female	245 (59.9)	46 (18.8)	–			
**BMI**						
<18.5	4 (1.0)	–	–			
18.5–24.9	130 (31.8)	22 (16.9)	–			
≥25	275 (67.2)	64 (24.7)	1.6 (0.9–2.7)	0.07		
**Cholelithiasis severity**						
Cholelithiasis only	294 (71.9)	43 (14.6)	–			
With cholecystitis	115 (28.1)	47 (40.9)	4.0 (2.5–6.6)	<0.0001	2.8 (1.5–5.1)	<0.001
**Comorbidity ICD−9–CM codes** ^**a**^						
Present	48 (11.7)	12 (25.0)	1.2 (0.6–2.4)	0.6		
Absent	361 (88.3)	78 (21.6)	–			
**Charlson comorbidity index**						
0	315 (77.0)	58 (18.4)	–			
≥1	94 (23.0)	32 (34.0)	2.3 (1.4–3.8)	<0.01	1.9 (1.0–3.5)	0.05
**Hospital admission**						
Urgency	77 (18.8)	19 (24.7)	1.2 (0.7–2.1)	0.5		
Planned	332 (81.2)	71 (21.4)	–			
**First operator**						
Resident surgeon	289 (70.7)	52 (18.0)	–			
Experienced surgeon	118 (28.9)	38 (32.2)	2.2 (1.3–3.5)	<0.005		
Missing	2 (0.5)	–	–			
**Intraoperative technical difficulties** ^**b**^						
Present	205 (50.1)	70 (34.1)	4.8 (2.7–8.2)	<0.0001	3.6 (1.8–6.2)	<0.001
Absent	204 (49.9)	20 (9.8)	–			
**Need of an additional trocar**						
Yes	115 (28.1)	45 (39.1)	3.5 (2.1–5.8)	<0.0001	2.5 (1.4–4.4)	<0.005
No	292 (71.4)	45 (15.4)	–			
Missing	2 (0.5)	–	–			
**Operative time**						
≤60 min	105 (25.7)	3 (2.9)	–			
>60 min	304 (74.3)	87 (28.6)	13.6 (4.2–44.1)	<0.0001		
**Estimated blood loss**						
≤10 ml	231 (56.5)	3 (12.1)	–			
>10 ml	178 (43.5)	62 (34.8)	3.9 (2.3–6.4)	<0.0001	3.0 (1.7–5.3)	<0.0001

*LC, laparoscopic cholecystectomy; SD, standard deviation; BMI, body mass index*.

a *Described in Table 1*.

b *Defined as fibrotic adherence, intentional puncture to decompress the gallbladder, gallbladder rupture with bile contamination of the operating field, bleeding from the cystic artery, difficult individuation of the anatomical structures, liver steatosis/cirrhosis, and subtotal cholecystectomy*.

At multivariate analysis in Table 3, cholecystitis (OR: 2.8, 95% CI: 1.5–5.1; p < 0.001), CCI (with no age) value ≥1 (OR:1.9, 95% CI: 1.0–3.5; p = 0.05), intraoperative technical difficulties (OR: 3.6, 95% CI: 1.8–6.2; p < 0.001), need of an additional trocar (OR: 2.5, 95% CI: 1.4–4.4; p < 0.005), and estimated blood loss >10 ml (OR: 3.0, 95% CI: 1.7–5.3; p < 0.0001), were the predictive factors that drove the surgeons to surgical drain placement after LC.

## Discussion

This study highlights how the surgical drain is used in a small number of patients undergoing unconverted LC from a purely descriptive point of view. In fact, in the patients under consideration, a surgical drain was placed in only 22% of the cases. This reflects the careful selection of the patient to use this device, according to what is widely reported in the current literature (5, 6, 8, 13). In their study, Park et al. (5) underline how the placement of intraoperative surgical drain has proved useful only in cases of difficult ligation of the cystic duct or hemostasis of the operative field and in the case of a suspected biliary leak. Conversely, they did not show a statistically significant difference in the onset of infected intra-abdominal collections between the group of patients with and without surgical drain after LC for acute cholecystitis. They conclude that the surgical drain should not be used prophylactically during LC because it does not reduce the morbidity and does not prevent local complications (such as, surgical site infection); rather it could be a risk factor for their onset (5). Qiu and Li (20) and Picchio et al. in their meta-analysis (6), have reported an increased number of complications, such as fever, wound infections, hemorrhage, wound herniations, increased postoperative pain, and hospital stay in patients with surgical drain placement. In our study, intraoperative drain placement was more frequent in older male subjects with BMI ≥ 25. This is particularly interesting considering that older age, male sex, and obesity are associated with a more severe acute cholecystitis clinical presentation (7, 21–25). Among the clinical conditions, the choice to place surgical drain correlates with the greater severity of the disease, particularly with the diagnosis of cholecystitis. This evidence is in agreement with what has been reported in the meta-analysis by Picchio et al. (6). Besides, these patients are clinically more complex, as expressed by their CCI (with no age). Both conditions put this group of subjects at a higher risk of perioperative complications, often the reason for the decision to use the drain at the end of surgery (26, 27). The emergency admission to the hospital has not shown to be significant in the decision to place surgical drain. Probably, the correct selection of the patient undergoing surgery, in accordance with the 2018 Tokyo Guidelines (28, 29), plays a key role. For example, in the case of fragile patients with emergency admission, it is preferable to adopt a conservative treatment with percutaneous drainage and antimicrobial therapy scheduling elective surgery subsequently, reducing the risk to place a drain (30–34). However, the results of the study by Ferrarese et al. (31), while confirming the safety and feasibility of LC in elderly patients both in the elective and emergency regimes, showed a statistically significant increase in the placement of surgical drain in patients operated in the emergency regime.

In the present study, the first operator is associated with the surgical drain placement after LC. In particular, residents place fewer drains than experienced surgeons. However, we believe that this association is more than a difference in management itself due to the patient selection. Indeed, the experienced surgeon attends during the surgery of residents, partake in the decision-making process, and takes the lead in the most complex intraoperative scenario. Therefore, experienced surgeons are the first surgeon for surgeries with a greater risk of intra- and postoperative complications, as demonstrated in the literature (19, 35, 36). Indeed, a greater complexity of the procedure, identified with the need to place an additional trocar, the finding of estimated blood losses >10 ml or a longer operating time, correlates with the choice to place the surgical drain after laparoscopic cholecystectomy in our study. At the univariate analysis, the potential preoperative predictors for a possible drain placement were found to be as follows: age over 64, male sex, diagnosis of cholecystitis, and a CCI (with no age) score ≥1. In their study, Bawahab et al. (15) show how the clinical condition, the preoperative complications, and the diagnosis of cholecystitis have a statistically significant correlation with the abdominal drain placement at the end of a LC. On the other hand, no study found an association between the CCI and abdominal drain placement during an LC. Among the intraoperative variables, as we have already mentioned, the need to insert an additional trocar, the presence of estimated blood loss >10 ml, and the operative time exceeding 60 min have been confirmed as predictors for the surgical drain placement. According to our bibliographic research, while increased operative time has already been correlated with the increased frequency of surgical drain use (15), no studies have reported the same evidence regarding the use of the additional trocar and intraoperative estimated blood loss. In particular, the diagnosis of cholecystitis, presence of intraoperative technical difficulties, need of an additional trocar, and an estimated blood loss >10 ml are found to be independent predictive factors for surgical drain placement in the multivariate analysis. The overall postoperative complications and those specifically related to surgery (wound infections, abdominal collections, biliary leaks, and incisional hernias) have shown a statistically significant correlation with the intraoperative placement of surgical drain. This correlation is confirmed by several sources in the literature (6, 13). For example, Tzovaras et al. (16) report how drain placement is associated with an increased frequency of postoperative pain and biliary leakage; therefore, its routine use in all elective cholecystectomies is not indicated. This aspect finds confirmation in Lewis et al. (37), who showed that no patient without surgical drain required reoperation or subsequent drainage procedure for subhepatic collections formation, as well as no difference in the frequency of wound infections. However, in other studies, such correlations are not confirmed with the statistical consistency (5, 20). As a result of our study, patients undergoing LC with surgical drain placement have a longer length of hospital stay than those without the placement of this device, as confirmed in previous studies (5, 6). In addition, if our study did not compare costs, a longer length of hospital stay associated with the use of surgical drain raise an economic issue as both hospitalization and materials drive cost.

The main limitation of this study is to be non-randomized, unbalanced, and retrospective. In addition, number of patients and multi-surgeon decisions on drain placement could limit results of this study. However, this preliminary study aimed to describe predictive factors for surgical drain placement during an LC into the current practice. Defining the predictive factors is the first step in identifying the target population for a future prospective study to evaluate the drain placement. The results of this study need to be carefully interpreted, according to the limitations and the aim of the study.

In conclusion, the placement of intraoperative drain after LC is done in selected patients as per the decision of the leading surgeon, with the theoretical purpose of early detection and minimizing complications. At the multivariate analysis, cholecystitis, CCI (with no age) ≥1, intraoperative technical difficulties, the need for an additional trocar, and estimated blood loss >10 ml were the predictive factors for surgical drain placement during LC. Otherwise, surgical drain placement is often associated with increased complications, length of stay, and re-intervention. Since studies showing high-quality evidence in patients with these characteristics are lacking, randomized prospective studies are needed to define the use of surgical drain during an LC in these specific populations.

## Data Availability Statement

The raw data supporting the conclusions of this article will be made available by the authors, without undue reservation.

## Author Contributions

GC and PB contributed to the study conception, study design, data acquisition, data interpretation, and manuscript drafting. RQ contributed to the study design, data acquisition, data analysis, and manuscript drafting. VB contributed to the study conception, study design, data interpretation, and manuscript drafting. All authors contributed to the article and approved the submitted version.

## Funding

Open access funding was provided by University of Udine.

## Conflict of Interest

The authors declare that the research was conducted in the absence of any commercial or financial relationships that could be construed as a potential conflict of interest.

## Publisher's Note

All claims expressed in this article are solely those of the authors and do not necessarily represent those of their affiliated organizations, or those of the publisher, the editors and the reviewers. Any product that may be evaluated in this article, or claim that may be made by its manufacturer, is not guaranteed or endorsed by the publisher.
