# Supplementary material for: Predictive Factors for Drain Placement After Laparoscopic Cholecystectomy
Source: Front Surg. 2022 Feb 2;8:786158. doi: 10.3389/fsurg.2021.786158 (PMC8847274; doi:10.3389/fsurg.2021.786158)
Supplement: Supplementary file 1 [file Table_1.DOCX]

**Predictive factors for drain placement after laparoscopic cholecystectomy.**

**Giacomo Calini M.D. ^1^*, Pier Paolo Brollo M.D. ^1^*, Rosanna Quattrin M.D. ^2^, Vittorio Bresadola M.D. ^1 ID^**

**^1^** General Surgery Department and Simulation Center, Academic Hospital of Udine, Department of Medicine, University of Udine, Udine, Italy

**^2^** Department of Organization of Hospital Services, Academic Hospital of Udine, Udine, Italy

* shared first authorship

Corresponding author

Vittorio Bresadola

ORCID: 0000-0002-0098-3540

Associate Professor of Surgery, Program Director of the General Surgery Residency Training, Department of Medicine, University Hospital S. Maria della Misericordia, P.zza S. Maria della Misericordia, 15 33100 Udine ITALY

Tel. +39 0432 559538/57, fax. +39 0432 559562, e-mail: vittorio.bresadola@uniud.it

**Supplementary material 1.
ICD-9-CM diagnoses used to classify comorbidities related to cholecystectomy.**

| **COMORBIDITIES** | **ICD-9-CM CODES** |
| --- | --- |
| Diabetes | 250 |
| Obesity | 278.0 |
| Anaemia | 280-284, 285 (excluding 285.1) |
| Coagulation disorders | 286 |
| Other haematological disorders | 287-289 |
| Hypertension | 401-405 |
| Ischemic heart diseases | 401-405 |
| Conduction disorders and arrhythmias | 426, 427 |
| Heart failure | 428 |
| Other heart diseases | 391, 393-398, 420-425, 429.0-429.9, 0.93.2, 745, 746.3-746.6 |
| Cerebrovascular diseases | 430-438 |
| Vascular diseases | 440-448, 557, 557.1 |
| COPD or respiratory distress | 490-496, 518.81, 518.82 |
| Chronic kidney disease | 582, 583, 585-588 |
| Chronic diseases (liver, pancreas, intestines) | 571, 572, 577.1-577.9 |
